# Forecasting the absolute and relative shortage of physicians in Japan using a system dynamics model approach

**DOI:** 10.1186/1478-4491-11-41

**Published:** 2013-08-27

**Authors:** Tomoki Ishikawa, Hisateru Ohba, Yuki Yokooka, Kozo Nakamura, Katsuhiko Ogasawara

**Affiliations:** 1 Graduate School of Health Sciences, Hokkaido University, N12W5, Kita-ku, Sapporo 060-0812, Japan; 2 Department of Medical Management and Informatics, Hokkaido Information University, Nishi Nopporo 59-2 Ebetsu, Hokkaido 069-8585, Japan; 3 Medical Informatics, National Institute of Radiological Sciences, 4-9-1, Anagawa, Inage-ku, Chiba 263-8555, Japan; 4 Faculty of Health Sciences, Hokkaido University, N12W5, Kita-ku, Sapporo 060-0812, Japan

**Keywords:** Forecasting the number of physicians, Shortage of physician, Maldistribution, System dynamics

## Abstract

**Background:**

In Japan, a shortage of physicians, who serve a key role in healthcare provision, has been pointed out as a major medical issue. The healthcare workforce policy planner should consider future dynamic changes in physician numbers. The purpose of this study was to propose a physician supply forecasting methodology by applying system dynamics modeling to estimate future absolute and relative numbers of physicians.

**Method:**

We constructed a forecasting model using a system dynamics approach. Forecasting the number of physician was performed for all clinical physician and OB/GYN specialists. Moreover, we conducted evaluation of sufficiency for the number of physicians and sensitivity analysis.

**Result & conclusion:**

As a result, it was forecast that the number of physicians would increase during 2008–2030 and the shortage would resolve at 2026 for all clinical physicians. However, the shortage would not resolve for the period covered. This suggests a need for measures for reconsidering the allocation system of new entry physicians to resolve maldistribution between medical departments, in addition, for increasing the overall number of clinical physicians.

## Introduction

Health policy planning for human resources has become an internationally high priority [1]. In Japan, a shortage of physicians, who serve a key role in healthcare provision, has been pointed out as a major medical issue [2]. The Ministry of Health, Labour and Welfare (MHLW) reported that there is lack of absolute and relative numbers of physicians, as well as maldistribution among regions and medical departments. To ease the personnel shortage in Japan, the MHLW estimated that hospitals need a further 24 000 physicians [3].

It has been suggested that the policy of decreasing medical school enrolment quotas is a major factor behind the absolute shortage of physicians [4,5]. In 1982 and 1999, the policy was approved by the Japanese Government to prevent future oversupply. The medical school enrolment quota had decreased from 8 280 students in 1984 to 7 625 in 2007. However, the number of physicians per unit of population in Japan is low compared with other developed countries [6]. According to Organization for Economic Co-operation and Development (OECD) data, the number of medical doctors per 1 000 persons in Japan was 2.2 in 2008; this number was 2.4 in the US in 2009 and 2.7 in the UK in 2010. The number of physicians in Japan has always been lower than the OECD average. In 2008 the MHLW identified the physician supply issue, and recognized that there is not only maldistribution, but also a shortage of absolute numbers of physicians [7]. To address the shortage, the Japanese Government decided to immediately increase the medical student enrolment quota. The Democratic Party of Japan (DPJ), which was elected into government from 2009 to 2012, promised to increase the quota to 1.5 times what it was in 2009 in order to raise the number of physicians per unit of population to the OECD average level [8].

Maldistribution of physicians at both the regional and departmental level has been reported by the MHLW [3]. Regional maldistribution is identified as a factor contributing to the generation of areas that fail to meet the basic healthcare needs of their community. Maldistribution among medical departments causes insufficient provision of some medical care. In Japan, obstetrics/gynaecology (OB/GYN) and emergency departments are especially mentioned as being in need of more physicians than other departments. Specifically, the number of OB/GYN physicians is decreasing, and the department is facing future shortages [9]. In Japan, medical school graduates determine their own speciality and location, so physician maldistribution may worsen. To address regional maldistribution, a bonding scheme has been proposed to ensure that graduates work in specified regions. No comprehensive measures have been proposed to address medical department-level maldistribution, and it is at risk of worsening in the future.

The healthcare workforce policy planner should consider future changes in physician numbers. Effective planning for the future medical workforce has become increasingly necessary in Japan because of changes in and uncertainty of physician supply in the face of the populations requirement for their services [10]. Accurate forecasting of the workforce supply is essential for effective planning. Forecasting methods include the need-based model, the demand-based model and benchmarking [11-13]. These methods are limited by assumptions about changes in physician availability across incomparable communities or areas [11]. We argue that the forecasting approach used should be able to describe the dynamic variables involved in the physician supply system and have utility even if the needs of the community and health system differ.

The system dynamics approach permits the inclusion of a dynamic factor in the models. We modeled the physician supply system to estimate the future number and sufficiency level of clinical physicians and forecast the absolute number of physicians required. Furthermore, we compared the sufficiency levels between all clinical physicians and specialists to evaluate department-level maldistribution. The purpose of this study was to propose physician-supply forecasting methodology by applying SD modeling to estimate future absolute and relative numbers of physicians.

## Materials and methods

### Data collection

We obtained data on the number of physicians from the national surveys of physicians, dentists and pharmacists conducted from 1972 to 2008 by the MHLW [9]. We extracted data on physician numbers, age, gender, speciality, and type of workplace from the survey. Medical specialities were defined according to the MHLW classification. To account for increases in medical school enrolment, we obtained data from the Ministry of Education, Culture, Sports, Science and Technology (MEXT) [14]. In addition, we used data from the basic school survey carried out by MEXT to identify past graduation numbers, and trends in speciality choice after graduation [15]. Moreover, the retirement rate was calculated by trend data considering the age-sex pyramids of the medical population. Other data sources are summarized in Table 1.

**Table 1:** Data sources for the simulation model

**Variables**	**Data sources**
Current workforce in baseline year (2008)	The number of physician reported by the Ministry of Health, Labour and Welfare in 2008
Medical school quota (current)	Bulletin about scheme of increases medical school quota
Pass rate at the national examination	Announcement about national examination for medical doctors
Career option rate	The school basic survey carried out by the Ministry of Education, Culture, Sports, & Technology
Retirement rates	We set the constant rate from trend data for the numbers of physicans reported by the MHLW
(include: death, change of speciality)	
Population projection in Japan	Population projection for Japan
	(National Institute of Population and Social Security Research)
Selection speciality rates	Questionnaire survey about clinical training in 2010

### SD modeling approach

Our model was based on SD, a methodology and computer simulation modeling technique for framing, understanding and discussing complex issues and problems. The approach was developed by computer pioneer Jay W Forrester in the mid-1950s [16]. It addresses the flow of people, processes, materials and information by exposing a tenet of all complex systems - feedback loops. According to SD, these loops are the major drivers of system behaviour. This methodology also enables us to address non-linear SD, which governs many real-life phenomena [16]. The approach has been used in a variety of contexts, including human resources planning, to gain an understanding of a system with complex dynamic and nonlinear interacting variables [17,18]. Internal feedback systems are represented in the SD model. The model is an interlocking set of differential algebraic equations developed from a broad spectrum of relevant measured and experiential data [16].

We used specialized software, STELLA® ver. 8.1.1 (isee systems, Lebanon NH, USA) to implement the SD models [19].

### Causal loop and stock and flow

**Figure 1:**
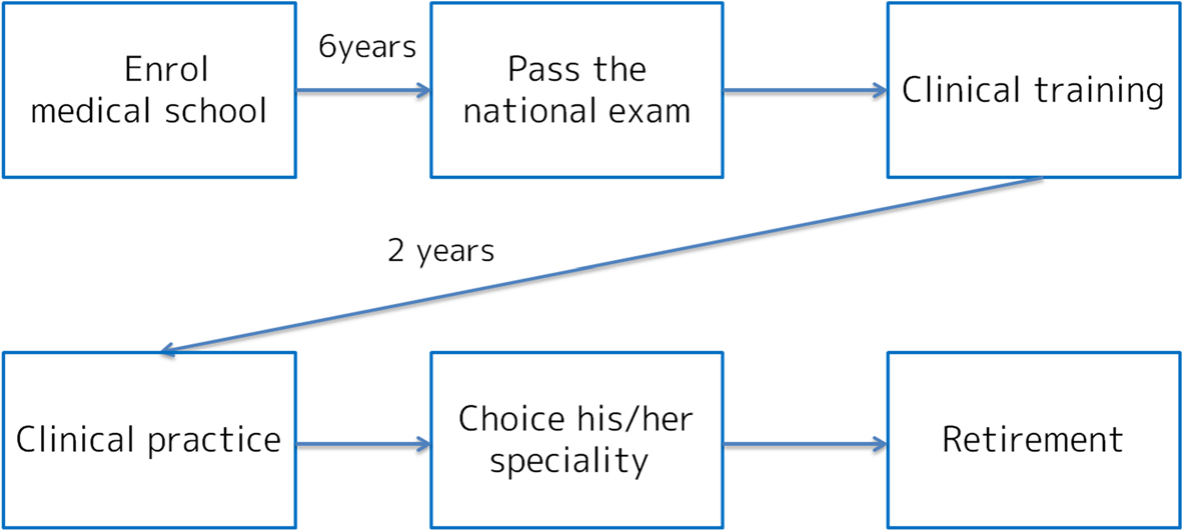
Conceptual scheme of the Japanese physician career path.

A causal loop diagram identifies the structures and interaction of feedback loops, and consists of variables for causal links. A causal link connects a cause variable with an effect variable by a link with a positive or negative change. To construct the physician supply model, we based the causal loop on the Japanese physician career path programme (Figure 1).

System accumulation is described by stock (or level) variables, such as the number of physicians, while the rate of change is described by flow variables, such as the annual number of physicians who retire. The net inflow determines the rate of stock change, that is, its time derivative. Therefore, the interaction between stock and flow is calculated mathematically by the following differential equation. In the equation, inflow and outflow denote the values of the inflow and outflow at any time between the initial time and the present time *t*[19].

(1) $$\frac{d\left(\mathrm{stock}\right)}{\mathrm{dt}}=\mathrm{Inflow}\left(t\right)-\mathrm{Outflow}\left(t\right)$$

### Uncertainty projection

Uncertainty in health projections must be assessed so that planners can anticipate possible variations and adapt the planning of human resources in consequence [10]. We conducted a sensitivity analysis to estimate the influence of the uncertainties of medical school quota and trends in graduate speciality choice on the forecast sufficiency level. We set the following quota and speciality choice scenarios from previous variables: increasing or decreasing the quota from the current number by 15%, and increasing or decreasing the number of graduates from the current number choosing OB/GYN by 1%.

### Criterion for evaluation the number of physicians

In planning the health workforce, a supply-need gap analysis must be conducted [11]. In Japan, there is no criterion for evaluating whether the number of physicians is sufficient or not. Conventionally, the number has been evaluated by a comparison with the OECD average level. In this evaluation, health workforce planners cannot account for the number of physicians that are required by patients or medical workers. We defined the physician number sufficiency level using data from the national survey carried out by the MHLW in 2010 to enable us to evaluate whether the future physician numbers are sufficient or not.

(2) $$\begin{array}{l} \text{sufficiency}\;\text{level}=\frac{\mathrm{the}\;\text{forecasted}\;\text{number}\;\mathrm{of}\;\text{physician}}{\mathrm{the}\;\text{required}\;\text{number}\;\mathrm{of}\;\text{physician}}\\ \;\times \text{corrective}\;\text{coefficient} \end{array}$$

In equation (2), the required physician number is derived from the claims of hospital administrators, and does not include clinics. The corrective coefficient corrects for missing clinic data and is the ratio of physicians who work in a hospital to those who work in a clinic.

In this study, if the sufficiency level is ≥1, we consider that the physician number is sufficient. When the sufficiency level is <1, we consider that there is a physician shortage. Furthermore, we evaluated speciality-level maldistribution by the difference in sufficiency level between all clinical physicians and OB/GYN physicians. If the sufficiency level of OB/GYN is <1 and the sufficiency level of all clinical physicians is ≥1 (and vice versa), we evaluated that there is speciality-level maldistribution between OB/GYN and other speciailties.

**Figure 2:**
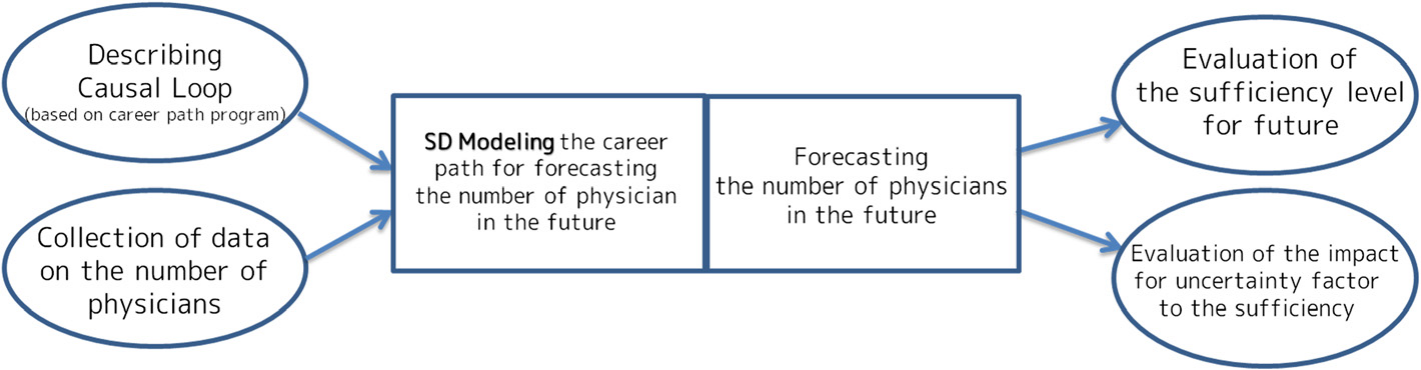
The system dynamics model methodology used in this study.

We constructed two models using the SD approach: (1) total number of clinical physicians, and (2) number of OB/GYN physicians. The base year was 2008 and the simulation is forecast up until 2030. The outcome measures were the numbers of clinical physicians and specialists (head count and per 1 000 persons) and sufficiency level. In order to test the validity of the models, we conducted a retrospective comparison of forecast and observed numbers in 1986 to 2008. The SD model methodology used for the health workforce forecasting in this study is displayed in Figure 2. The key model assumptions are summarized in Table 2.

**Table 2:** Key assumption of the simulation model

**Variable**	**Key assumptions**
New medical graduates	Domestic students only
	The number of graduates is equal to the medical student quota
Retirement	Retirement, deaths and movement speciality rates remain constant

### The validity of the SD model

The validity of model forecasts should always be tested. As a necessary step in SD methodology to validate the model, historical and simulated data should be compared for specific years [20]. We examined the simulated and observed physician numbers in 1998, 2000, 2002, 2004, 2006, and 2008. The method is to compare the simulation data and observed historic data, and calculate the relative and mean squared errors of the number of physicians:

(3) $$e=\frac{|({ŷ}_{t}-y_{t})|}{y_{t}}$$

(4) $$\mathrm{MSE}=\sqrt{\frac{\sum_{1}^{n}e_{t}^{2}}{n}}$$

Here, *y*_*t*_ represents the observed number of physicians in the year *t*; ${\hat{y}}_{t}$ represents the simulated number of physicians in the year *t*; *e* is the relative error of the number of physicians and mean squared error (MSE) is the mean of the squares of the relative errors.

## Results

**Figure 3:**
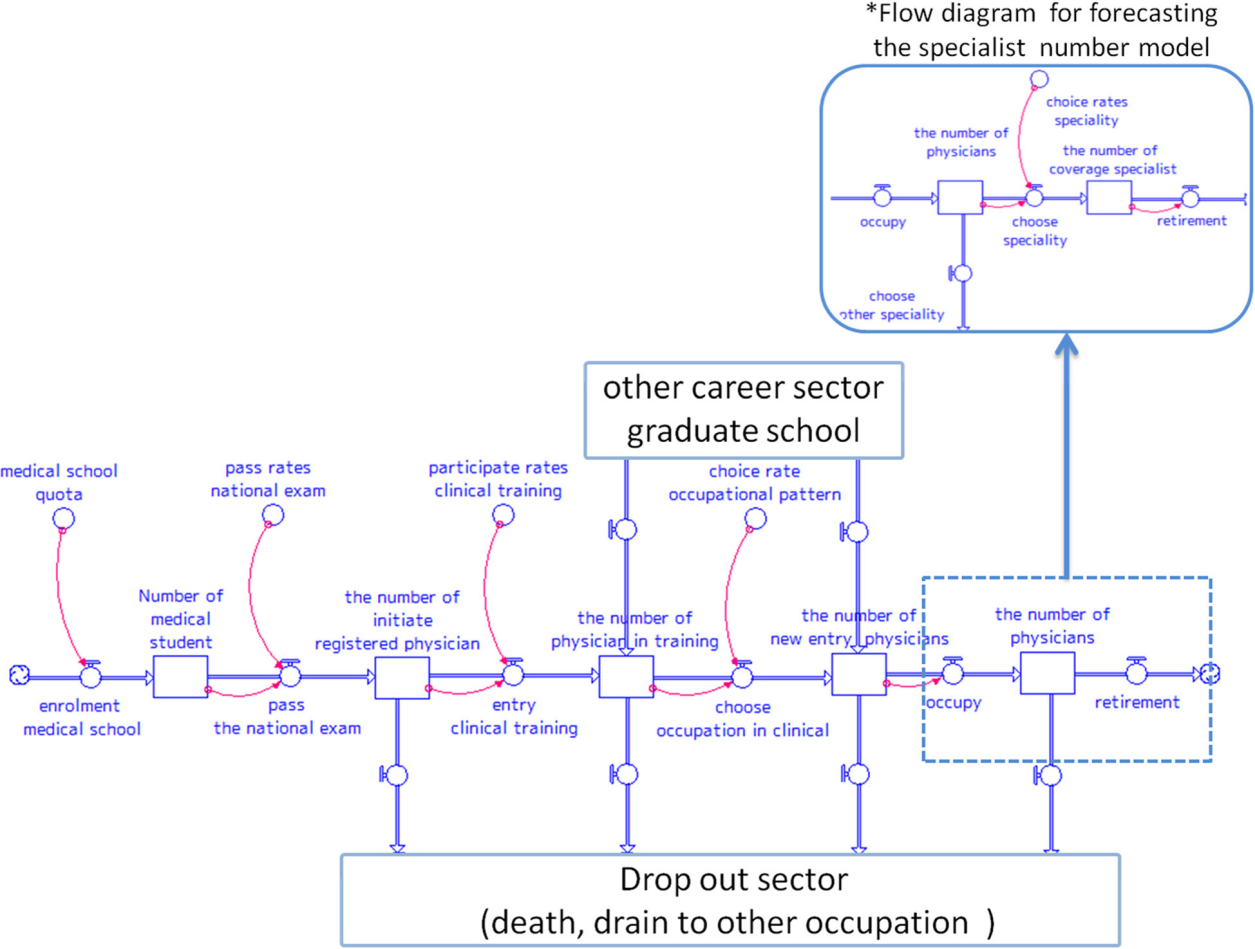
Diagram of the system dynamics model for forecasting the number of clinical physicians.

**Figure 4:**
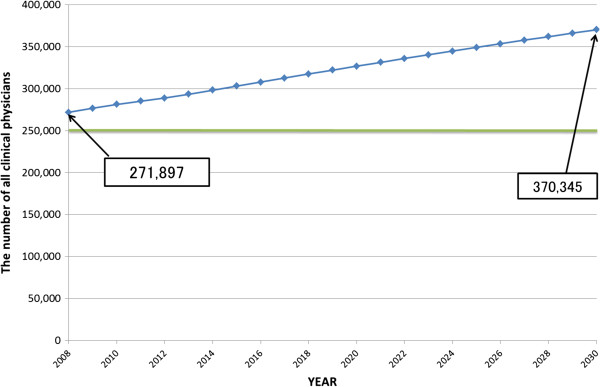
**Forecast numbers of all clinical physicians, 2008 to 2030.**

**Figure 5:**
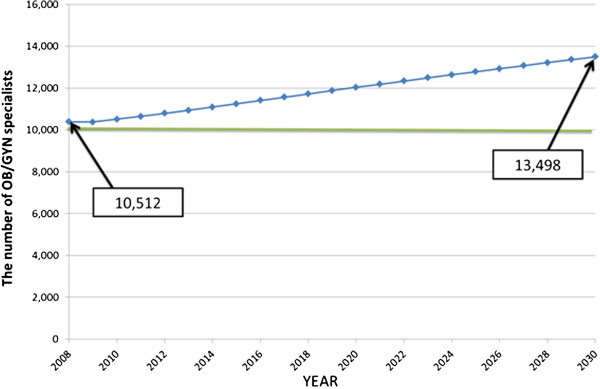
**Forecast numbers of obstetrics/gynaecology specialists, 2008 to 2030.** OB/GYN, obstetrics/gynaecology.

**Figure 6:**
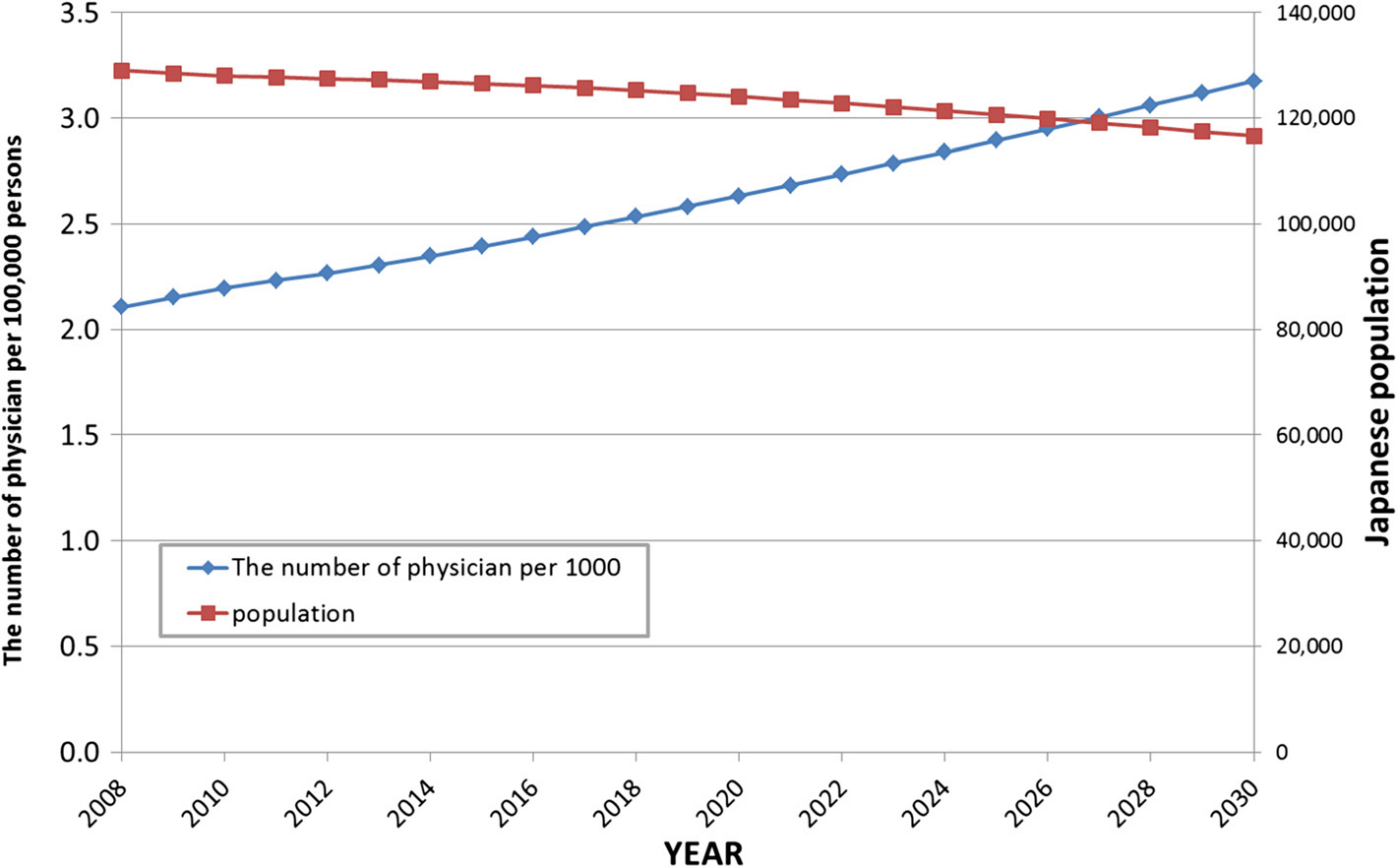
Forecast numbers of all clinical physicians per 1 000 persons.

**Figure 7:**
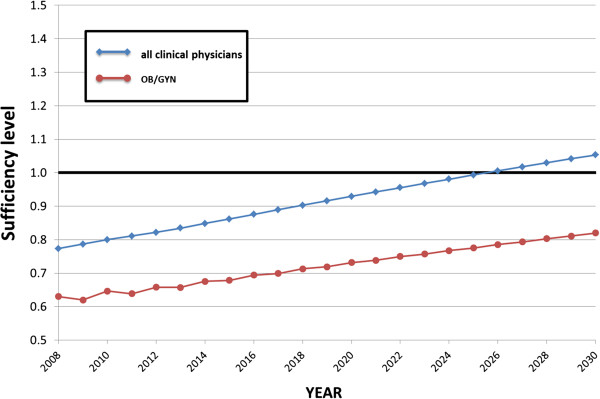
Forecast sufficiency level, 2008 to 2030.

Figure 3 displays the causal loop and stock and flow diagrams of the SD model. There are six main sectors in this diagram; enrolment, national examination, clinical training, clinical practice, choice of speciality, and retirement. Figures 4 and 5 display the forecast numbers of all clinical physicians and OB/GYN specialists. In 2008, there were 271 897 clinical physicians in Japan’s medical workforce. Of these, 10 388 were OB/GYN specialists. The number of clinical physicians was forecast to increase by 370 345 during 2008 to 2030. This represents an average annual growth rate of 1.6%. Figure 6 displays the forecast number of physicians per 1 000 persons and the population projection for Japan by the National Institute of Population and Social Security Research in 2012. The number of physicians per 1 000 persons was 2.2 in 2008, and was forecast to increase by 3.18 per 10 00 persons during 2008 to 2030. This represents an average annual growth rate of 2.2%. Figure 7 displays the forecast sufficiency level. The number of clinical physicians was in shortage in 2008. However, it was forecast to gradually increase and reach a sufficient level by 2026. The number of OB/GYN specialists was forecast to increase by 13 498 by 2030. The forecast sufficiency level of OB/GYN specialists was less than one during the entire period 2008 to 2030. Therefore, we evaluated that there would be speciality-level maldistribution. The sufficiency level growth rate for all clinical physicians was 36.21% during the forecast period. On the other hand, the rate for OB/GYN specialists, who are especially required, was only 30.1%. The sufficiency level of all clinical physicians surpass one, while the sufficiency level of OB/GYN specialists would not have reached one after 2026.

**Figure 8:**
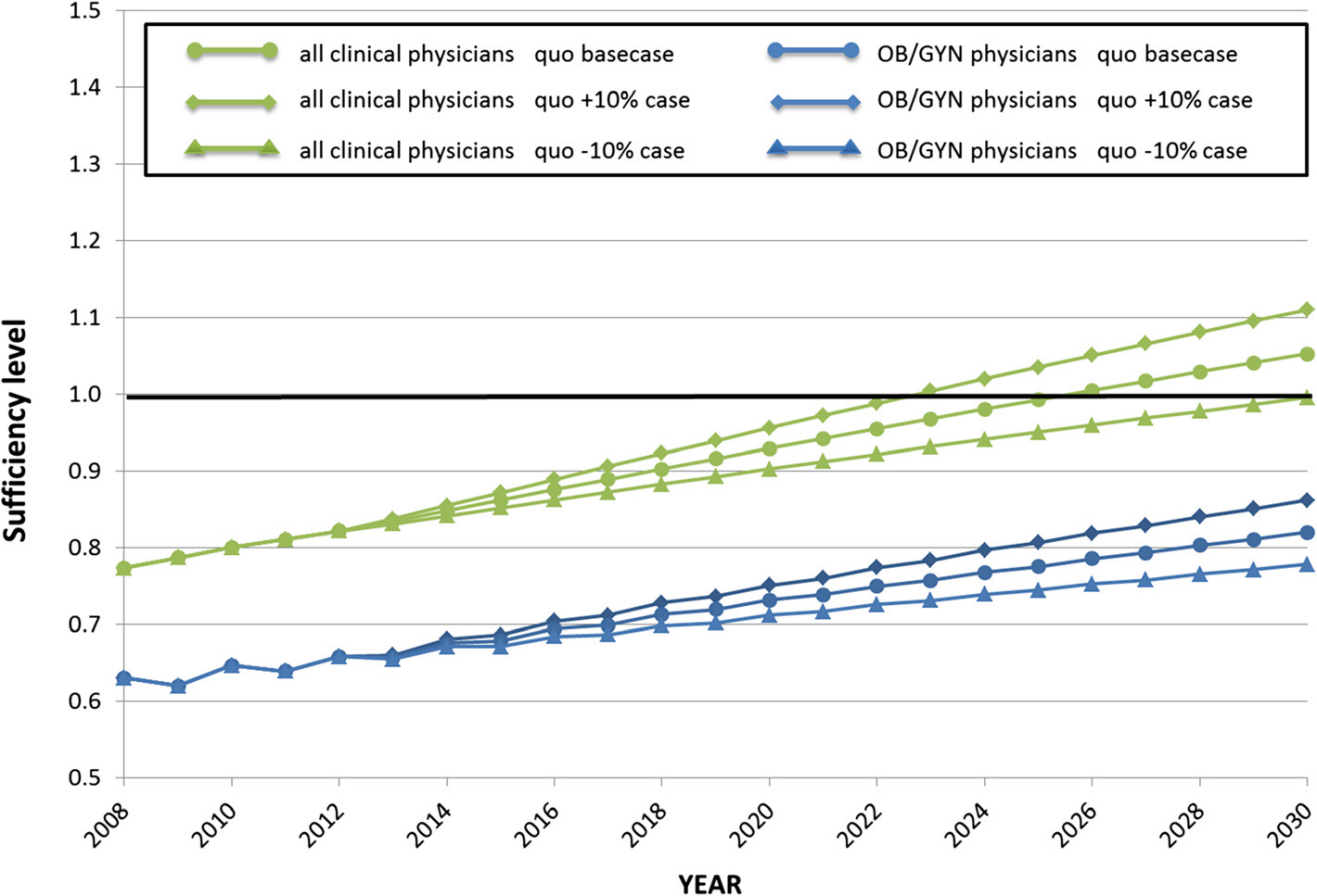
**Sensitivity analysis of the sufficiency level of all clinical physicians and obstetrics/gynaecology specialists, 2008 to 2030.** OB/GYN, obstetrics/gynaecology; quo, quota.

**Figure 9:**
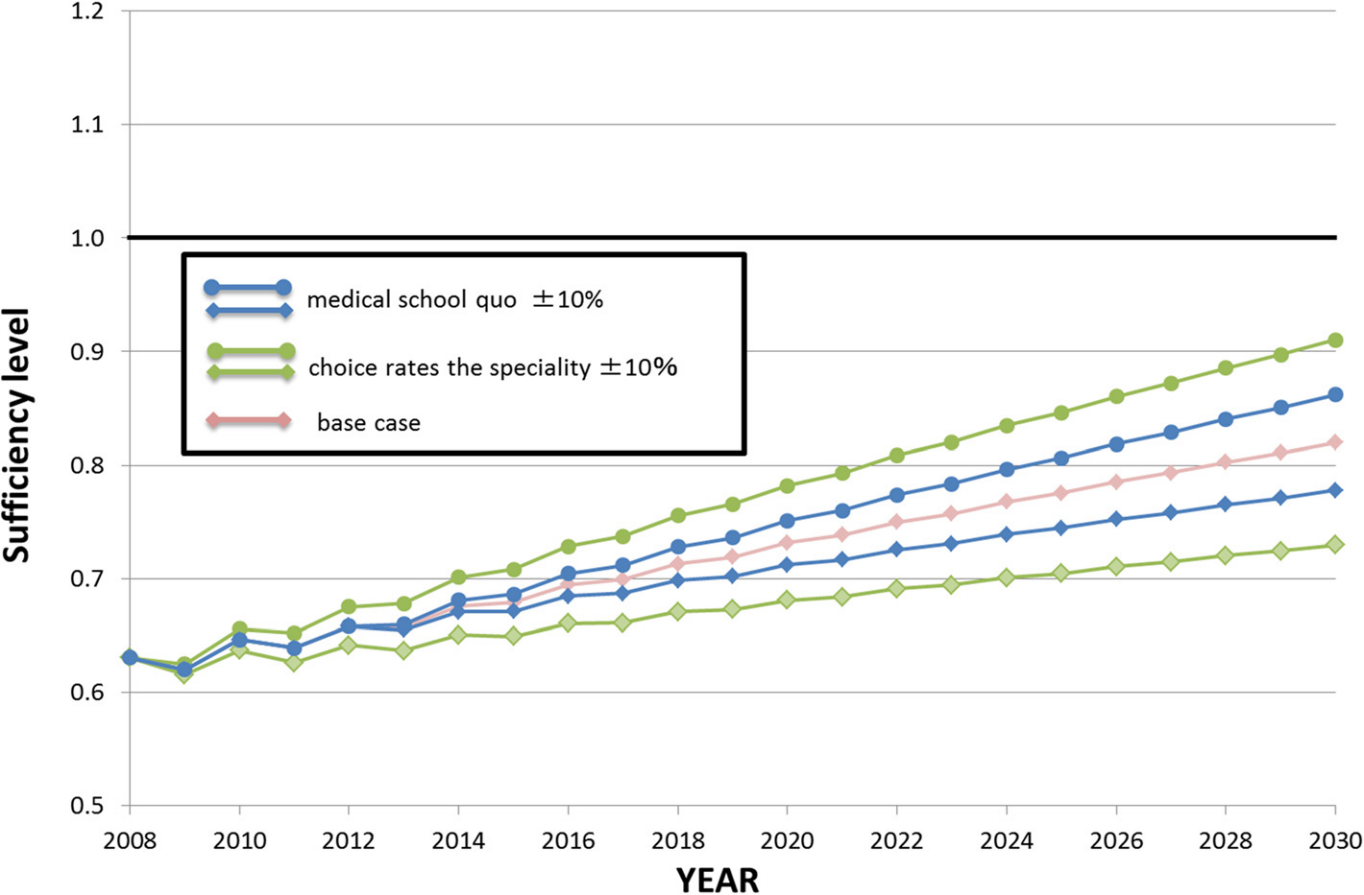
Comparison of impact for medical school quota and speciality choice on sufficiency level.

The results of the sensitivity analysis are shown in Figures 8 and 9. The analysis of all clinical physicians under the increased quota scenario shows an accelerated growth of sufficiency levels from shortage to sufficient. In the decreasing quota scenario, the growth of sufficiency levels was delayed compared with the baseline scenario, and did not reach one. For OB/GYN specialists, neither the increased nor decreased quota scenario leads to a sufficient level (Figure 8). When comparing the influence of the medical quota versus the speciality option, the latter had a more significant impact on the sufficiency level than the former (Figure 9).

Simulated and observed historical data for specific years were compared to validate the SD model. Table 3 presents this comparison as well as the calculated MSE.

**Table 3:** The comparison of historic data and simulation data

**Year**	**1998**	**2000**	**2002**	**2004**	**2006**	**2008**	**MSE**
Historic data	248.61	255.79	262.68	270.37	277.93	286.7	0.007
Simulation data	250.39	257.88	264.74	271.16	277.09	283.71	
Relative error	0.007	0.008	0.008	0.003	0.003	0.01	

## Discussion

Our model indicates that the number of clinical physicians in Japan will continue to grow during 2008 to 2030. However, under the baseline scenario, a sufficient number of physicians would not be reached until 2026. The DPJ promised in its manifesto to raise the number of physicians per unit of population to the OECD average level. We determined that the number of clinical physicians per 1 000 persons would not surpass the OECD average until 2029 (the OECD average was 3.1 in 2008; our forecast number is 3.12 by 2029). Our analysis shows that the DPJ promise would be fulfilled within the years covered in the SD model.

Our model forecasts that the shortage in the absolute number of clinical physicians would be resolved during the coverage timespan of the SD model. However, the number of OB/GYN specialists would still remain in shortage in 2030. Given from above, we evaluated that there would be speciality-level maldistribution. We speculate that the difference in the sufficiency-level growth rate raises/expands the maldistribution. We propose that measures for increasing the numbers of OB/GYN specialists is required in addition to measures for increasing the overall number of clinical physicians.

The sensitivity analysis provided an evaluation of the impact of each factor. Figure 8 shows that the number of physicians would be sufficient in 2023 in the increased medical student enrolment quota scenario (+15%), whereas it would remain in shortage in 2030 in the decreased medical student enrolment quota scenario (−15%). Figure 9 shows that increasing the number of graduates choosing OB/GYN has a greater impact on the OB/GYN sufficiency level than increasing the medical student enrolment quota for OB/GYN.

The sensitivity analysis, looking from different angles, can be interpreted as a scenario analysis. In Japan, most physicians build their career as indicated in Figure 1. With the matter in mind, the rates of speciality choices of potentially causes an increased shortage of the number of specialists, as shown in Figure 9 under the −2% scenario. In this way, even if the absolute shortage is resolved, the uncertainty of variation in choices of speciality possibly causes shortages in specific speciality, and the speciality-level maldistribution. Medical school graduates voluntarily determine their speciality in Japan. This generates the uncertainty of variation in the rates of speciality choices. Therefore, we highlight the necessity of strategic planning for speciality options, for example, by obligating a proportion of medical school enrollees to choose a specific department.

The validity of the model forecasts should always be tested. Our model was validated by calculating the relative error and MSE from simulated and observed historic data. Generally, MSE <10% is considered reasonable [20]. According to equations (3) and (4), our MSE was calculated as 0.007 <0.1 (Table 3). Furthermore, the relative error had no effect on our evaluation of the number of physicians. We evaluated the impact of relative error as being very low, and therefore concluded that our model was valid.

Some previous studies and the government have also estimated the numbers of these professionals by different methods. But these estimates have been based on the static model method. We performed the estimate considering the dynamic change of some value that impacts the physician number. As with any modeling exercise, our model was constructed under the assumption the system would not change. A change in the physician supply system would require further modeling. Therefore, we should be rethink the forecasting model structure when the system changes.

In this study, we defined a criterion for judging whether the number of physicians is sufficient or not. The criterion was calculated based on the survey performed by the MHLW in 2010 [3]. The data were collected using a self-report questionnaire for the medical institutions. They subjectively provided their required number of physicians in 2010, judging from daily clinical practice. Therefore, an evaluation using the required number of physicians from the survey would be based on the assumption that answers to the questionnaire reflect regional need. We assume the required number reflect health statistics, morbidity rate and prevalence rate and so on, based on their honest answers. In the OB/GYN domain, in particular, the female-specific influences of fertility rate and the incidence of gynaecological carcinoma for instance, was a consideration in the OB/GYN specialists’ opinions. The required physician number by MHLW 2010 did not provide the demographics of age or gender. Therefore, the criterion does not reflect a difference between genders. We need to explore the quantification method considering the difference. Moreover, the criterion cannot take account of the impact of dynamic change on need. Generally, as time goes on, it is expected that the required number of physicians will change. We need to account for this change when estimating the sufficient number of physicians needed in the future.

Our model did not account for the shortage or maldistribution between regions. SD modeling permits the addition of a physician transfer factor to the model. By adding this factor, the forecast number of physicians would enable us to evaluate the future supply-need balance while focusing on the presence or absence of regional or departmental shortages. We demonstrated that the SD modeling approach can be used to forecast changes in the number of physicians and the sufficiency level. The model provided two key suggestions. First, the absolute shortage of physicians will resolve during the forecast period because the number per population would reach the 2009 OECD average and the sufficiency level would reach one. Second, the number of OB/GYN specialists is at risk of remaining in shortage. Measures to address the uncertainty in medical trainees’ choices of speciality are necessary to prevent an increase in department-level maldistribution. Obligating a proportion of medical school enrollees to choose a specific department will remove this uncertainty.

A proactive approach to the planning and management of the medical workforce, involving continued monitoring, would facilitate more frequent and less dramatic adjustments to supply. This in turn would reduce the likelihood of extreme supply conditions (shortages or surpluses) and assist in more effective delivery of medical care. SD modeling enabled us to analyze multiple perspectives, considering the causal links, factor uncertainties, and different scenarios. However, this study has some limitations derived from the difficulty in obtaining some data. Increasing the collection of data on physician supply and the required number of physicians would minimize these limitations.

## Conclusion

In this study, we defined a criterion for judging whether the number of physicians is sufficient or not. The criterion was calculated based on the survey performed by the MHLW in 2010 [3]. Generally, as time goes on, it is expected that the required number of physicians will change. We need to account for this change when estimating the sufficient number of physicians in the future. Our model did not account for the shortage or maldistribution between regions. SD modeling permits the addition of a physician transfer factor to the model. By adding this factor, the forecast number of physicians would enable us to evaluate the future supply-need balance while focusing on the presence or absence of regional or departmental shortages. We demonstrated that the SD modeling approach can be used to forecast changes in the number of physicians and the sufficiency level. The model provided two key suggestions. First, the absolute shortage of physicians will resolve during the forecast period because the number per population would reach the 2009 OECD average and the sufficiency level would reach one. Second, the number of OB/GYN specialists is at risk of remaining in shortage. Measures to address the uncertainty in medical trainees’ choices of speciality are necessary to prevent an increase in department-level maldistribution. Obligating a proportion of medical school enrollees to choose a specific department will remove this uncertainty. A proactive approach to the planning and management of the medical workforce, involving continued monitoring, would facilitate more frequent and less dramatic adjustments to supply. This in turn would reduce the likelihood of extreme supply conditions (shortage or surplus) and assist in more effective delivery of medical care. SD modeling enabled us to analyze multiple perspectives, considering the causal links, factor uncertainties, and different scenarios. However, this study has some limitations derived from the difficulty in obtaining some data. Increasing the collection of data on physician supply and the required number of physicians would minimize these limitations.

## Abbreviations

DPJ: Democratic Party of Japan; MEXT: Ministry of education, culture, sports, science and technology; MHLW: Ministry of health, labour and welfare; MSE: Mean squared error; OB/GYN: Obstetrics/gynaecology; OECD: Organization for Economic Co-operation and Development; SD: System dynamics.

## Competing interests

The authors declare that they have no competing interests.

## Authors’ contributions

TI performed the investigation. TI analyzed the data. TI wrote the manuscript. TI, YY, HO, KN, and KO interpreted the data and contributed substantially to its revision. KO conceived the study, and participated in its design and coordination and helped to draft the manuscript. All the authors read and approved the final manuscript.
